# From Retention Time to Functional Group Assignment: A Chemical Database‐Driven Approach for High‐Resolution Mass Data of Marine Dissolved Organic Matter

**DOI:** 10.1002/rcm.10043

**Published:** 2025-04-14

**Authors:** Fabian Moye, Marlo Bareth, Boris P. Koch, Jan Tebben, Tilmann Harder

**Affiliations:** ^1^ Marine Chemistry, Faculty of Biology and Chemistry University of Bremen Bremen Germany; ^2^ Department of Ecological Chemistry Alfred‐Wegener‐Institut Helmholtz Zentrum für Polar‐ und Meeresforschung Bremerhaven Germany; ^3^ Faculty of Mathematics and Computer Science University of Bremen Bremen Germany; ^4^ University of Applied Sciences Bremerhaven Germany

**Keywords:** chemodiversity, DOM, LC‐FT‐MS, octanol–water coefficient, structure prediction

## Abstract

**Rationale:**

The high chemodiversity of marine dissolved organic matter (DOM) has challenged identification of singular DOM components. To infer chemical structure features of DOM with accurately determined molecular formulas, we assessed if empirically determined elution properties of stoichiometrically identical isomers obtained from a chemical database would predict features, such as the type and number of functional groups, in structurally unknown DOM isomers.

**Methods:**

DOM of different origin (North Sea, Southern Ocean) was analysed by LC‐FT‐MS using two different mass spectrometry methods. Chromatographic retention of DOM isomers was correlated with calculated retention properties of structurally known isomers in PubChem. A total of 7.7 million chemical identifiers were queried for their computed octanol–water coefficients (logP). The 50 most intense molecular formulas were queried for PubChem structure data files. The number and type of structural features was assigned to logP bins across the DOM elution window, to correlate the contribution of database‐derived structure features to retention time of structurally unknown DOM isomers.

**Results:**

The intensity‐weighted average of logP for C_x_H_y_O_z_ in Southern Ocean water, predicted by retention time of all molecular formulas, was in good agreement with logP values stored in PubChem. The comparatively longer retention of the same isomer in North Sea versus Southern Ocean DOM suggests a decoration with fewer alcohol groups and more ring structures and esters. Earlier eluting molecular formulas are more likely to contain rings and alcohols, while later eluting ones are more linear/branched and contain more esters.

**Conclusions:**

We hypothesised DOM isomers belonging to comparatively older Southern Ocean water to elute earlier than young and less degraded molecules present in North Sea water. This hypothesis was verified based on three exemplarily selected molecular formulas cooccurring in all water samples. Our strategy circumvents issues of chimeric fragmentation spectra and furthermore adds retention time as a new physicochemical descriptor of DOM molecules.

## 1 Introduction

Marine dissolved organic matter (DOM) is among the most complex mixtures of organic molecules on earth [1, 2]. Typical mass spectra of DOM contain a wide spectrum of different molecular formulas (ca. 3000) in the mass range of *m/z* 200 to *m/z* 700, each of which has at least 30 isomers [1, 2], resulting in at least 90 000 different compounds. The chemical composition and molecular diversity of DOM results from both the duration and repetition of biogeochemical transformations (e.g. microbial activity, mixing, physicochemical transformations, aggregation [3, 4, 5]) of primary produced organic matter [6, 7]. Consequently, the chemical composition and diversity of DOM is an imprint of its environmental origin, history and fate. For example, the stoichiometric composition differs between marine and terrestrial DOM, with the former being more aliphatic and richer in nitrogen, and the latter mainly derived from lignin, more aromatic and poorer in nitrogen [5]. Diverse reactions lead to chemical diversification of DOM, e.g. oxidative de‐aromatisation of phenolic molecules [8]. In combination with cycloadditions [8], these reactions increase the molecular weight and stoichiometric ratio of O/C and consequently alter the type and number of functional groups. During transport and aging in the open ocean, DOM is oxidised and decreases in molecular weight [9, 10, 11].

These transformations do not only result in a plethora of different molecular formulas, but importantly, the same molecular formula may also result from different sources and biogeochemical and physicochemical inputs [1, 7]. In other words, the historical environmental impact on the plethora of organic molecules results in distinguishable abundances of isomers, i.e. compounds sharing the same molecular formula and mass but having different structural arrangements of atoms or groups.

DOM is typically analysed by direct injection mass spectrometry, mainly with Fourier‐transform ion cyclotron resonance MS (FT‐ICR‐MS) [12] or high‐resolution Orbitrap mass spectrometry (HR‐MS) [13, 14]. To avoid ionisation artefacts and ion suppression, DOM is increasingly analysed in hyphenation to liquid chromatography (LC) [15, 16, 17, 18]. Yet, even under LC‐MS conditions, the enormous structural diversity of DOM results in total ion chromatograms (TICs) with broad peak shape and without baseline resolution. This is due to the fact that individual isomers elute as a continuum of overlapping gaussian peaks along the entire polarity gradient [1]. Notably, this phenomenon is not caused by single compounds ‘smearing’ across the chromatogram, but rather due to the plethora of individual chromatographically resolved structural isomers that together result in one unresolved continuous mass detector signal spanning the entire chromatographic retention time window. This explanation agrees with the observation that extracted ion chromatograms of internal standards spiked into a complex DOM matrix still reveal very sharp gaussian peaks [19].

The elemental ratios of DOM constituents obtained by either one of the above analytical methods serve as valuable proxies for biochemical and physiochemical processes related to origin and diagenetic fate of DOM molecules. A number of qualifiers are calculated from the molecular formulas derived from high‐resolution accurate mass spectrometry to characterise and distinguish different DOM molecules by stoichiometry, origin and transformation state, e.g. double‐bond equivalents (DBEs), nominal oxidation state of carbon (NOSC) [20] and the aromaticity index (AI) [21, 22]. While these qualifiers are useful to characterise and distinguish between different molecular formulas, they are mostly unable to distinguish between isomers. Moreover, the high inherent structural diversity of DOM has so far challenged the separation and structural identification of singular DOM components [23], and only very few DOM molecular structures are known (e.g. Arakawa et al. [10], Seidel et al. [24], Geuer et al. [25], Papadopoulos Lambidis et al. [26]).

In this paper, we tested if we could correlate the chromatographic retention of a priori structurally unknown DOM isomers with their decoration by functional groups that largely affect their chromatographic properties. This hypothesis was inspired by the observation that extracted ion chromatograms of internally spiked standards of known accurate mass indeed revealed sharp retention despite coelution among isomers within the DOM mixture [15]. This in turn suggested that different chromatographic behaviour (i.e. retention time) of structurally unknown molecules could be useful to predict structure motifs that govern their chromatographic behaviour, such as typical functional groups (alcohols, aldehydes, esters, carboxylic acids, ethers, alicyclic and aromatic rings). We hypothesised that the position of narrow retention time bins across the DOM chromatogram would indirectly offer chemical structure information of DOM isomers, such as the type and number of functional groups decorating these isomers.

Chromatography theory posits the three‐dimensional structure and surface charge distribution on any molecule to largely govern its equilibrium between adsorption on stationary versus solubility in a mobile phase, and hence result in chromatographic separation of structurally different analytes. The octanol–water coefficient (K_OW_ or P; commonly log‐transformed as logP) describes the liquid partitioning between a non‐miscible apolar and polar phase and thus approximates the thermodynamic equilibrium on chromatography columns [16, 27, 28]. It has been successfully used to predict chromatographic retention times [16, 29, 30]. The coefficient logP is determined either experimentally or predicted computationally, e.g. with the XLOGP3‐algorithm [31]. To test the conceptual idea outlined above, we theoretically correlated the chromatographic retention of accurate mass‐derived molecular formulas of structurally unknown DOM isomers with curated structure database information of known isomers having the same molecular formula.

In this study, we analysed three DOM samples of different origin, including coastal North Sea water and shallow and deep Southern Ocean water, by LC‐FT‐MS. We correlated the chromatographic retention of individual DOM isomers in these samples with calculated chromatographic retention properties of structurally known isomers obtained from a compound database. Briefly, molecular formulas of DOM constituents in each sample were annotated and the assigned molecular formulas searched in PubChem (https://pubchem.ncbi.nlm.nih.gov/). More than 7.7 million chemical identifiers were filtered and queried for logP entries. The molecular formulas were ranked by intensity, and the 50 most intense molecular formulas of each water sample were queried for PubChem structure data files resulting in a dataset of 11 237 structural isomers with known features, such as alcohols, aldehydes, esters, carboxylic acids, ethers, alicyclic and aromatic rings. The distribution of the number and type of these structural features was assigned to logP bins across the DOM sample elution window in order to correlate the contribution of database‐derived structure features to the retention time of structurally unknown DOM isomers. Three of the 50 most intense molecular formulas were exemplarily chosen to test if the same isomers in comparatively young (North Sea) and old (Southern Ocean) DOM could be distinguished by chromatographic retention. Given that more recalcitrant and older DOM is generally more transformed and structurally modified by polar functional groups, such as carboxylic or hydroxy functions [10, 32, 33], we hypothesised the DOM isomers belonging to comparatively older Southern Ocean water to elute earlier than young and less degraded molecules present in North Sea water.

## 2 Material and Methods

### 2.1 Samples

Two samples of the Weddell Sea (latitude −67.5633°, longitude −55.3448°) from different depths representing marine (30 m depth) and refractory DOM (1356 m depth) were obtained as described elsewhere [34, 35]. Briefly, 160 L Antarctic seawater were sampled with a rosette sampler on RV Polarstern during ANT XXII/2. The water was filtered with 0.2 μm filter cartridges, acidified to pH 2 and pumped through 60 mL solid phase extraction cartridges (PPL, 5 g). DOM was eluted with 40 mL methanol and stored at −18 °C. Fresh coastal DOM is extracted routinely by us from the Southern North Sea (latitude 54.1447°, longitude 7.8711°) and used as laboratory standard. Sea water was sampled over 0.2 μm PTFE (Whatman) filter on RV Uthörn, acidified to pH 2 and extracted with PPL cartridges. DOM was eluted with methanol and extracts were stored at −18 °C until measurement to minimise esterification in methanol [36].

### 2.2 Liquid Chromatography and Mass Spectrometry

While FT‐MS is well established, comparative interlaboratory experiments have shown that identified molecular formulas are not well reproduced between different instrument platforms [37]. Therefore, we chose to analyse the data with two instruments to confirm that identified analytical trends were reliable across different analytical platforms.

All samples were analysed on two instruments: (a) Fourier Transform Orbitrap mass spectrometer (FT‐Orbitrap‐MS; Q‐Exactive Plus, Thermo Fisher Scientific, Bremen, Germany) coupled to ultra‐high performance liquid chromatography (UPLC, Vanquish, Thermo Fisher Scientific, Bremen, Germany), herein referred to as LC‐FT‐Orbitrap‐MS, and (b) 7 T scimaX MRMS system (Bruker Daltonics GmbH & Co. KG, Bremen, Germany) coupled to UPLC (Elute LC, Bruker Daltonics GmbH & Co. KG, Bremen, Germany), herein referred to as LC‐FT‐ICR‐MS. Reversed‐phase chromatography was done with a C18 column (Waters AQUITY 2 x 100 mm, 1.7 μm) at 0.3 mL min^−1^ and a linear gradient of A (ultrapure water, 4 mmol L^−1^ ammonium formate) 2 min, 99%; 11 min, 0%; 14.9 min, 99%; B (MeOH, 4 mmol L^−1^ CHOONH_4_) 2 min, 1%; 11 min, 100%; 14.5 min, 100%; 14.9 min, 1%.

LC‐FT‐Orbitrap‐MS was performed using heated electrospray ionisation (HESI‐II, Thermo Fisher Scientific, Bremen, Germany) in negative mode with a spray voltage of 2.5 kV, capillary temperature of 256 °C, sheath gas flow of 47.5 and auxiliary gas flow rate of 11.3 (arbitrary units). Full MS mode with a resolution of 280 000 (*m/z* 200) and a scan range of *m/z* 150 to *m/z* 1875 was used. The ion optics and ESI source settings were tuned to maximise the intensity of the peak at *m/z* 400. Each scan was internally calibrated with nine lock masses (339.10854, 369.11911, 411.12967, 469.13515, 541.15628, 595.20323, 611.19814, 651.22944, 693.24001). The Xcalibur software package (Thermo Electron Corporation) was used to export the mass lists, intensity, noise level and resolution to individual peak lists.

LC‐FT‐ICR‐MS were acquired with a capillary voltage of 4 kV. Drying gas temperature was set to 220 °C, Neb gas flow to 7 L min^−1^ and drying gas pressure to 2 bar. Measurements were performed using electrospray ionisation in negative ion mode with a mass resolution of 640 000 (*m/z* 200) using 4 M data points. The scan range was set to *m/z* 107 to *m/*z 2000, and eight single scans were added. The spectra were online lock mass‐calibrated with the formate adduct of Hexakis(1H,1H,2H‐perfluoroethoxy)phosphazene at *m/z* 666.019887. The method parameters were adjusted for maximum intensities at *m/z* 400. DataAnalysis 5.3 software package (Bruker Daltonics GmbH & Co. KG, Bremen, Germany) was used to export the mass lists, intensity and mass resolution of the added scans every 1.1 min.

### 2.3 Elution Properties of Standard Compounds in a DOM Sample

Twenty‐five standard substances (see Table S1) were measured under the same chromatographic conditions to determine their retention time and chromatographic peak width. Subsequently, 12 standard substances (Table S2) were selected by their ionisation efficiency in negative ESI and spiked to the North Sea water sample. Their concentrations were adjusted to achieve an intensity of ≈10^6^, a common intensity range for DOM peaks in the LC‐FT‐Orbitrap‐MS.

### 2.4 Annotation of Molecular Formulas

Scans were averaged to time bins of 1 min to reduce data size and enhance S/N ratios. Each scan was externally calibrated with the Ultra‐Mass‐Explorer (UME, https://gitlab.awi.de/bkoch/ume; Leefmann et al. [38]) using a linear calibration and 839 known DOM molecular formulas as calibrants. Molecular formulas were annotated using UME with the following elemental composition: ^12^C_≤∞_^1^H_≤∞_^16^O_≤∞_^14^N_≤2_^32^S_≤1_. The mass accuracy was 0.3 ppm for LC‐FT‐ICR‐MS and 0.8 ppm for LC‐FT‐Orbitrap‐MS.

The following molecular properties were calculated: DBEs, NOSC according to LaRowe et al. [20] with neutral charge and zero phosphorus and the aromaticity index [21, 22]. The index of degradation (I_DEG_, Flerus et al. [11]) was calculated if all 10 molecular formulas were present. Similarly, the terrestrial index (I_*terr*_, Medeiros et al. [39]) was calculated if all 80 molecular formulas were present. Molecular formulas were filtered by a DBE‐*O* (DBE minus number of oxygen atoms) to maximum of 10 [40], and molecular formulas were only considered when a ^13^C or ^34^S isotope mass peak was present. Formulas identified as part of a list of potential surfactants [38, 41] were removed from the data set and the blank subtracted. The LC‐FT‐ICR‐MS data did not contain any multiple assignments, while 0.12% of the LC‐FT‐Orbitrap‐MS peaks matched either C_x_H_y_O_z_ or C_x_H_y_O_z_N_1–2_S_1_ (x, y and z stand for any possible number within the boundaries of annotation). Multiple assignments were filtered by $\min\left(|\mathrm{DBE}-\#O|\right)$ [40]. The absolute intensities were converted to relative intensities by sum normalisation so that the sum of all intensities per bin was 1. Detailed equations are reported in S1.1. Ion chromatograms were extracted by converting the LC‐FT‐Orbitrap‐MS files into .mzML with proteowizzard (version 3.0.22353) [42] and using the R API of MassQL [43].

### 2.5 Calculation of Octanol–Water Coefficients

The octanol–water coefficient is defined as the partitioning ratio of a substance in octanol ($c_{O}$) and water ($c_{W}$).

$$K_{\mathrm{OW}}=P=\frac{c_{O}}{c_{W}}$$

With the logP as:

$$\log_{10}P=\log_{10}\left(\frac{c_{O}}{c_{W}}\right)=\log_{10}c_{O}-\log_{10}c_{W}$$

LogP is not routinely measured; therefore, in databases like PubChem, logP values are predicted computationally. Calculated logP values, according to the XLOGP3‐algorithm [31], are referred to as XlogP. The assigned molecular formulas were searched in the PubChem database (https://pubchem.ncbi.nlm.nih.gov/) and queried for XlogP entries. More than 7.7 million chemical identifiers (CIDs) were found and filtered to remove entries containing two molecules or peroxide structures. The 50 most intense molecular formulas detected by the mass spectrometer within all three DOM samples (LC‐FT‐ICR‐MS) were additionally queried for their structure data files (sdf). After removing peroxides 11 580 sdf of the 50 most intense molecular formulas remained.

### 2.6 Data Evaluation

Data were evaluated using R [44]. Structure‐data files queried from PubChem were analysed with ‘ChemmineR’ [45]. Recursive feature elimination (random forest, ‘ranger’ package [46, 47], 1000 trees, 5 times repeated cross‐validation, 5 folds) was used to find relevant properties for the prediction of XlogP. Further statistics were reported according to Greenacre [48].

## 3 Results

### 3.1 Chromatographic Peak Width as a Measure of Chemodiversity

**FIGURE 1:**
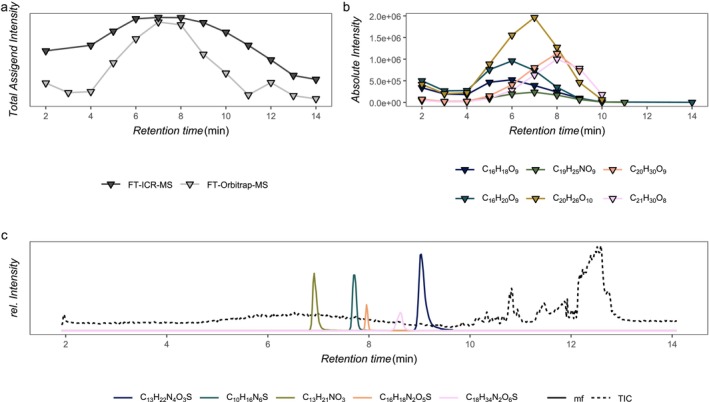
(a) Total assigned intensity (TAI) of the North Sea water standard for LC‐FT‐ICR‐MS and LC‐FT‐Orbitrap‐MS. Scans were averaged into 1 min bins (triangles). (b) Extracted ion chromatograms of the LC‐FT‐Orbitrap‐MS for typical DOM formulas had the characteristic broad elution peak (up to 6 min). Exemplary molecular formulas were chosen to represent different sections of the chromatogram (early (C_16_H_18_O_9_, C_16_H_20_O_9_, RT 4 to 6 min), mid (C_19_H_25_NO_9_, C_20_H_26_O_10_, RT 6 to 7 min) and late (C_20_H_30_O_9,_ C_21_H_30_O_8_, RT 7 to 11 min) phase of the chromatogram. (c) The North Sea water standard was spiked with additional standard substances: ranitidine (C_13_H_22_N_4_O_3_S, 9.0 min), cimetidine (C_10_H_16_N_6_S, 7.7 min), salbutamol (C_13_H_21_NO_3_, 6.9 min), phenoxymethyl penicilin (C_16_H_18_N_2_O_5_S, 8.0 min) and lincomyacin (C_18_H_34_N_2_O_6_S, 8.6 min). These standards were chosen by their elution maxima and their ionisation efficiency in negative ESI. Their concentration was adjusted so that each compound had an intensity maximum in a range of a typical DOM peak (absolute intensity ≈10^6^). The spiked standards had elutions windows of about 0.1 to 0.4 min. For a detailed list of all 25 spiked standards with the respective intensity and retention time, see Table S1. The mass tolerance of the extracted ion chromatograms was 1 ppm for the pharmaceutical standards and 0.8 ppm after external calibration for DOM molecular formulas.

Under the chosen chromatographic conditions (see above), DOM eluted from 4 to 13 min. The total assigned intensity (TAI) showed a maximum at 7 min with both methods, LC‐FT‐ICR‐MS and LC‐FT‐Orbitrap‐MS (Figure 1a). Exemplary molecular formulas revealed the known broad DOM elution profile across several minutes, spanning the complete elution gradient (4 to 10 min, Figure 1b). However, elution maxima differed between different molecular formulas: early C_16_H_18_O_9_, C_16_H_20_O_9_ (RT, 4 to 6 min), mid C_19_H_25_NO_9_, C_20_H_26_O_10_ (RT, 6 to 7 min) and late C_20_H_30_O_9,_ C_21_H_30_O_8_ (RT, 7 to 11 min) (Figure 1b). For comparison, standard substances spiked to a North Sea water DOM sample had an elution window of 0.1 to 0.4 min, typical for UHPLC applications (Figure 1c).

**FIGURE 2:**
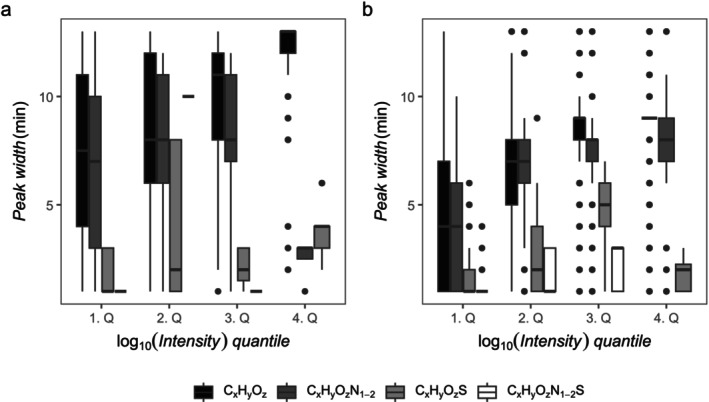
(a) LC‐FT‐ICR‐MS, (b) LC‐FT‐Orbitrap‐MS. The chromatographic peak width (1 min bins) of all molecular formulas of the North Sea sample correlated with the number of heteroatoms and the signal intensity. (a) Intensity quantiles: 1. Q, < 10^6.46^; 2. Q, 10^6.46^–10^6.75^; 3. Q, 10^6.75^–10^7.13^; 4. Q, > 10^7.13^; (b) 1. Q, < 10^4.31^; 2. Q, 10^4.31^–10^4.80^; 3. Q, 10^4.80^–10^5.23^; 4. Q, > 10^5.23^. The shallow and deep Southern Ocean sample showed similar results (Figure S4).

The chromatographic peak width of DOM molecular formulas correlated with their intensity (Figures 2, S3 and S4). The molecular formulas were separated into four quantiles (1. Q, 25%; 2. Q, 50%; 3. Q, 75%; 4. Q, 100%) based on their log‐transformed intensity (base 10). Overall, C_x_H_y_O_z_ molecular formulas had the highest intensities and peak widths. The peak width distribution for all quantiles spread across the complete elution gradient. The North Sea sample was chosen as an example, as the shallow and deep Southern Ocean samples behaved similarly (see Figure S5). C_x_H_y_O_z_ molecular formulas (x, y and z represent any possible number of atoms within the annotation range) had the broadest elution window of up to 13 min. In the lowest intensity quartile, their elution window differed between 1 min and 13 min, while in the highest intensity quartile all C_x_H_y_O_z_ molecular formulas ranged between 8 and 13 min peak width. C_x_H_y_O_z_N_1–2_ molecular formulas behaved similarly on both instruments, but only four molecular formulas occurred in the highest intensity quartile for LC‐FT‐ICR‐MS (LC‐FT‐Orbitrap‐MS: 255 molecular formulas). Ninety‐seven C_x_H_y_O_z_S and C_x_H_y_O_z_N_1–2_S molecular formulas were detected by LC‐FT‐ICR‐MS. They mostly occurred in the North Sea sample (North Sea: 39, Shallow SO: 30, Deep SO: 28) with a peak width between 1 and 3 min in the first quartile and between 1 and 6 min in the fourth quartile. By LC‐FT‐Orbitrap‐MS, we detected 814 C_x_H_y_O_z_S and C_x_H_y_O_z_N_1–2_S molecular formulas distributed over all samples (North Sea: 294, Shallow SO: 263, Deep SO: 257). Their peak width did not steadily increase but peaked in the third quartile in the North Sea sample with an elution window between 1 and 9 min for C_x_H_y_O_z_S.

### 3.2 Comparison of Empirical Elution Profiles With Properties Mined From the Chemical Structure Database PubChem

The XlogP values of 27 standards measured under the same conditions were queried in PubChem (Table S8, Figure 4a). After removing C_26_H_29_NO (tamoxiphen) and C_22_H_24_N_2_O_8_ (tetracycline) as outliers, there was a significant linear relationship between the XlogP value and the retention time ($\begin{array}{c} \text{XlogP}=\\ 0.58t_{r}-3.72,F_{1,24}=6.28,R_{\mathrm{adj}}^{2},p-\text{value}:<0.001,\text{standard error}::\\ \left(\text{intercept}\right)\;0.92,\left(\text{slope}\right)\;0.10 \end{array}$) confirming good prediction of chromatographic behaviour.

The molecular formulas detected by LC‐FT‐ICR‐MS were ranked by intensity, and the top 50 molecular formulas were selected (Tables S4 and S5). The same formulas were detected by LC‐FT‐Orbitrap‐MS. Their elution maxima ranged from 2 to 12 min (median 7 min). Their mean molecular O/C ratio was 0.4799(0.0467), their mean molecular H/C ratio 1.2843(0.0925), their mean DBE was 8 (1) and their mean *m/z* 427 (33). The selected molecular formulas were queried on PubChem (https://pubchem.ncbi.nlm.nih.gov/) for structure‐data file (sdf) information, which resulted in a dataset of 11 237 structural isomers after removing peroxides and adducts.

**FIGURE 3:**
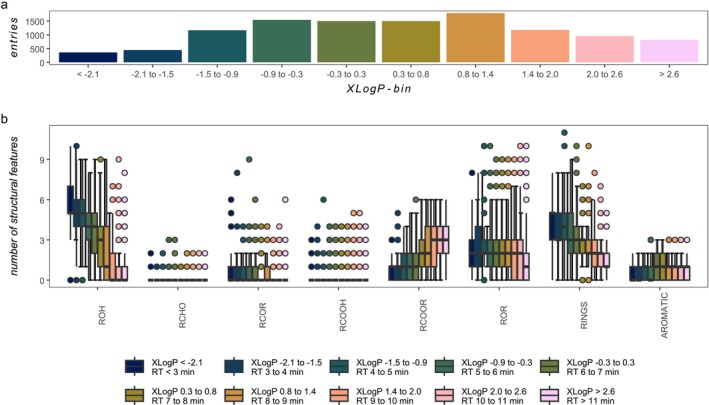
Structural features of 50 selected molecular formulas in PubChem (11 237 structures). (a) Number of PubChem entries across the different XlogP bins. (b) Distribution of the number of structural features (points mark outlier) by XlogP bin. The legend shows the XlogP bins and the respective retention time window (for equation, see text).

DOM molecules predicted to elute first (XlogP < −2) contained more rings (median 4 [2.5% quantile, 2; 97.5% quantile, 6; *n* = 422]), ketones (median 0 [0, 2, *n* = 422]) and alcohols (median 6 [3, 8, *n* = 422]) groups, while late eluting DOM molecules (XlogP > 3) were predicted to be more esterified (median 3 [0, 5, *n* = 482]) and aromatic (median 1 [0, 2, *n* = 482], Figure 3, Tables S6 and S7). The recursive feature elimination (random forest, 1000 trees, 5 times repeated cross validation, 5 folds) selected the following molecular properties and functional groups as most important to predict the XlogP (in this order): R‐OH, aromatic structures, molecular weight, number of rings, R‐O‐R, RCOR (*R* ^2^ 0.81, RMSE 0.63). Aldehydes and carboxylic groups contributed less to the prediction of XlogP because they did not vary along the elution gradient.

**FIGURE 4:**
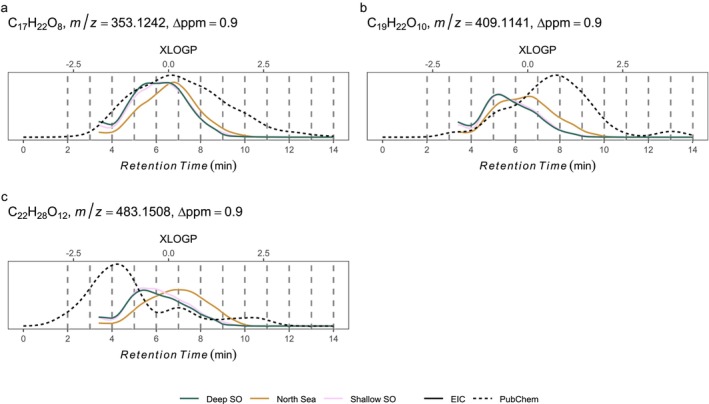
Extracted ion chromatograms of three exemplary molecular formulas differed in retention depending on sample origin (solid lines). The isomeric assemblage behind the selected molecular formulas, as found in PubChem (dashed line), covered the DOM elution gradient of the EICs (LC‐FT‐Orbitrap‐MS, spline smoothed, first 100 scans were cut‐off). *m/z* is given for [M‐H]^−^.

The chosen PubChem molecular formulas covered the complete elution gradient of DOM molecular formulas (Figures 3 and 4). Each of the 50 molecular formulas revealed a different PubChem‐obtained isomeric assemblage behind these formulas (Figure S5). Three exemplary extracted ion chromatograms (EIC) with a good overlap of available PubChem isomers present in North Sea and Southern Ocean samples were analysed in detail (C_17_H_22_O_8_, C_19_H_22_O_10_, C_22_H_28_O_12_) and showed shorter retention in both Southern Ocean compared to the North Sea water sample (Figure 4).

### 3.3 Linear Correlation Between Empirical Retention and Theoretical logP for all Molecular Formulas

Based on the linear regression, retention times of all molecular formulas were converted to logP and average‐weighted by their intensity, i.e. the chromatographic peak maximum had the highest weight. The correlation between them and the average XlogP value found in PubChem was tested for each sample and heteroatom group, with a high correlation indicating a good match to PubChem entries. The correlation was tested with a weighted least‐square regression with the residuals as weights to reduce sensitivity to outliers. C_x_H_y_O_z_ in the Southern Ocean samples had the highest correlation (slope: Shallow SO: 0.86, *p* < 0.001, Deep SO: 0.90, *p* < 0.001, North Sea: 0.48, *p* < 0.001). While C_x_H_y_O_z_N_1–2_S and C_x_H_y_O_z_S molecular formulas had no significant linear relationships in SO samples, significant linear relationship was observed for C_x_H_y_O_z_N_1–2_ (slope: Shallow SO: 0. 57, *p* = 0.01, Deep SO: 0.56, *p* = 0.03, North Sea: 0.22, *p* = 0.13; Table S3). The North Sea sample had a significant correlation to all heteroatom types but with variation in strength (C_x_H_y_O_z_ 0.48, C_x_H_y_O_z_N_1–2_‐0.10, C_x_H_y_O_z_N_1–2_S 0.17, C_x_H_y_O_z_N_1–2_S‐0.11; all *p* < 0.001).

## 4 Discussion

To characterise DOM isomers in marine water extracts, we correlated their computed chromatographic retention with molecular structure features, such as their assignment with functional groups that largely determine the chromatographic retention. With a set of three exemplary DOM isomers, we assessed if the empirically determined elution properties of stoichiometrically identical isomers obtained from a chemical compound database would predict structural features, such as the type and number of functional groups, in structurally otherwise unknown DOM isomers.

To test this conceptual idea, we first verified if extracted ion chromatograms of internally spiked standards of known accurate mass indeed revealed sharp retention despite coelution among isomers within the DOM mixture. We screened 24 commercially available organic compounds that mimic the stoichiometry of DOM constituents in the range of C_16_H_18‐20_O_9_ (early elution), C_19‐20_H_25‐26_N_0‐1_O_9–10_ (medium elution) 6 to 7 min) and C_20‐21_H_30_O_8–9_ (late elution). Spiking the North Sea DOM sample with 12 selected standards did indeed result in narrow peaks of each standard (Figure 1), supporting previous findings by Patriarca et al. [19]. Subsequently, the DOM molecular formulas were ranked by intensity and the first top 50 molecular formulas were selected and queried on PubChem for structure data resulting in ca. 12 000 structural isomers including predicted XlogP values. The good linear relationship between XlogP values and retention time promised high predictive power. Among the identified molecular properties and functional groups, the presence of alcohols, aromatic structures, molecular weight, esters, rings and ketones correlated best with XlogP (rare feature elimination; Figure 3). Contrary, aldehydes and carboxylic groups did not support the correlation between XlogP and retention time, nor did these functional groups vary across the elution gradient. Irrespective of the analytical instrument used, the weighted average of predicted logP was a good linear predictor for the average XlogP of corresponding structures found on PubChem for C_x_H_y_O_z_ molecular formulas in both Southern Ocean samples, whereas the opposite was found in the North Sea sample. Thus, the coverage of structure types in PubChem was better for the Southern Ocean than the North Sea samples.

The diagenetic imprint in any given DOM molecule co‐occurring in water masses of different age or origin is caused by numerous reaction pathways that result in molecules of the same molecular formula but different decoration with functional groups [1, 7]. Aged DOM molecules are more oxidised and smaller [9, 10, 11] and thus more polar, resulting in weaker chromatographic retention. The extracted ion chromatograms of three exemplary DOM isomers indeed confirmed our hypothesis that a DOM isomer belonging to comparatively older Southern Ocean water elutes earlier than its counterpart in young and less transformed North Sea water (Figure 4).

Deep Southern Ocean DOM mainly consists of aged and oxidised molecules. Due to its short retention on a hydrophobic LC column, the oxygen is likely present in oxygen‐containing functional groups, such as alcohols (this study Figure 3, see also Arakawa et al. [10], Lam et al. [32]). This explanation is in analogy to prior studies demonstrating that weaker retained DOM molecules show high H_2_O loss in MS^2^ fragmentation experiments, indicative of high abundance of alcohol functions [1]. Conversely, the number of carboxylic groups along the DOM elution gradient, as indicated by neutral loss of CO_2_ in MS^2^, was shown to be rather constant and unaffected by retention time [1, 49]. These experimental observations strongly support our theoretical prediction (Figure 3). The comparatively longer retention of the same isomer in North Sea DOM thus suggests the decoration with fewer alcohol groups and more ring structures and esters. This chemical difference between both isomers is likely explained by riverine input of terrestrial DOM into the Southern North Sea, where it mixes with freshly produced marine DOM. Terrestrial riverine DOM, with its high phenolic content, offers many precursor molecules whose dearomatisation and cycloaddition will form complex ring structures with oxygen stored in esters, anhydrides or lactones rather than alcohols [8].

The analysis of three marine DOM samples (North Sea, shallow and deep Southern Ocean) on two different LC‐MS platforms revealed that chromatographic peak widths of extracted ion chromatograms correlated with the elementary composition of DOM molecular formulas, especially the number and types of heteroatoms (Figure 2). In terms of the elution window width, corresponding to the degree of chemodiversity of molecules in this time frame, the narrow and short elution of C_x_H_y_O_z_N_1–2_S stoichiometries demonstrated this category to have the lowest chemodiversity. Since marine primary production predominately results in autochthonous organic matter with high C:N ratios [50], this is congruent with the observed high chemodiversity.

**FIGURE 5:**
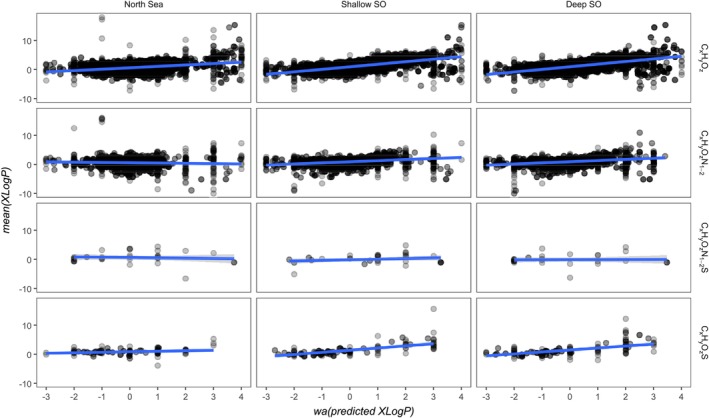
Based on retention time, the logP value of each molecular formula was predicted and intensity‐weight averaged. A linear model was constructed between these and the average XlogP value of all respective isomers available in PubChem. Depicted are all molecular formulas (*n* = 5048) detected by LC‐FT‐Orbitrap‐MS. A perfect fit (slope = 1, intersect = 0, $R^{2}\approx 1$) means that retention of the average DOM molecular formulas perfectly match the average XlogP value of PubChem structures. In this case, DOM structures would be predicted with high likelihood by structures of the same molecular formula stored in PubChem. The strength of the correlation varied between sample origin and heteroatom class. There were ca. 4 million XlogP entries for the LC‐FT‐ICR‐MS molecular formulas and ca. 7.6 million XlogP entries for the LC‐FT‐Orbitrap‐MS molecular formulas$\left(\text{residual weighted linear model},R_{\mathrm{adj}}^{2}=0.23,p<0.001,F_{23;46142}=603.5\right)$, further statistics including the model for LC‐FT‐ICR‐MS are summarised in Table S3.

The intensity‐weighted average of logP for the elementary composition C_x_H_y_O_z_ in Southern Ocean samples, predicted by retention time of all molecular formulas (Figure 5), was in good agreement with XlogP values stored in PubChem (slope close to 1). For the other elementary compositions under investigation (Figure 5), the relationship was weaker, i.e. the slope was closer to zero or not significantly different from zero. This probably means that the average composition of structures stored in PubChem only partially covered the average measured structural composition of DOM molecules. This does not contradict or invalidate our conceptual approach of combining empirical retention time of DOM isomers to theoretical logP values, and in turn decoration with functional groups, because PubChem compounds cover the complete elution gradient (Figure 4). The explained variance ($R_{\mathrm{adj}}^{2}=0.23$) highlights the wide range of compounds projected on a molecular formula. We propose our approach to be advantageous over direct‐injection mass‐based library searches [51] as well as fragmentation‐based annotation approaches [52, 53, 54], because our strategy circumvents issues of chimeric fragmentation spectra and furthermore adds retention time as a new physicochemical descriptor of DOM molecules. The combination of a constrained chemical structure feature space by retention time and fragmentation might facilitate targeted DOM fragmentation experiments, similar to Simon et al. [52].

Inter‐ and intra‐platform comparisons of mass spectrometric data often yield variable results in the direct comparison of detected molecular formulas and their intensity [14]. Such platform‐related differences are commonly assigned to different response factors, resulting from ionisation and ion transfer efficiencies prior to detection [57, 58]. However, multivariate comparisons have revealed comparable trends between instruments on the multivariate level and reproduced trends (this study Figures S1 and  S2) [37, 55, 56]. The data of this study are in good agreement with these results because assigned molecular formulas overlapped to ~31% between platforms. The percentage of the total intensity explained by the sum formulas found by both instruments was ~98% for LC‐FT‐ICR‐MS and ~73% for LC‐FT‐Orbitrap‐MS. Interestingly, FT‐Orbitrap‐MS detected more heteroatom‐containing molecules (Figure 2), which may be due to platform‐specific ionisation efficiency and/or a detection of more low intense signals (often heteroatom containing) due to different signal to noise cut‐off.

We are aware that prediction and classification of DOM structural features by XlogP requires careful consideration, because PubChem contains known compounds with unknown similarity to DOM, and extracted PubChem data predict XlogP based on the uncharged molecule. Having measured the DOM samples in this study at pH 4–5, we can only assume that carboxylic acids were largely undissociated. Yet, as detailed structures of DOM constituents are unknown, the exact protonation and charge state cannot be estimated, thus introducing a bias of unknown magnitude since the adsorption equilibria between solid and mobile phase, especially for carboxylic rich molecules, is pH‐dependent [26]. In this study, we only considered single negative charged ions. Future work will have to carefully examine the pH effect when predicting compound structure on the basis of calculated retention times.

## 5 Conclusion

By correlating empirical retention time data with theoretical octanol–water partition coefficients, we were able to predict the occurrence of specific functional groups in otherwise structurally completely unknown DOM molecules. We compared the partition coefficient with structural information stored in PubChem and could thus indicate the type and abundance of distinct functional groups in DOM molecules depending on their retention time. We verified that less retained DOM isomers on a C18‐LC column were likely to contain more alcohol groups and ring elements than stronger retained isomers, instead containing more esters and ethers. This approach circumvents the chimeric spectra of DOM MS fragmentation experiments. Using three exemplary DOM molecular formulas found in water samples of different origin and age, we demonstrated that the diagenetic state of these formulas aligned with changes in their retention time and thus with their decoration with different functional groups. We further demonstrated that DOM MS spectra obtained on two different LC‐MS platforms, namely LC‐FT‐ICR‐MS and LC‐FT‐Orbitrap‐MS, were robust and suitable for this conceptual approach. We also showed that chromatographic peak width of EICs belonging to a distinct elementary composition of DOM molecules was a good measure of their underlying chemodiversity.

## Author Contributions

**Fabian Moye:** writing – original draft, visualisation, formal analysis. **Marlo Barth:** investigation. **Boris Koch:** writing – review. **Jan Tebben, Tilmann Harder:** writing – review and editing, supervision.

### Peer Review

The peer review history for this article is available at https://www.webofscience.com/api/gateway/wos/peer‐review/10.1002/rcm.10043.

## Supporting information


**Figure S1** Screeplot of the principal component analysis, see Figure S2.
Figure S2 Principal component analysis (PCA) of the weighted averages (wa) of the bulk parameters (weights: absolute intensity) for each sample and retention time bin. (a) LC‐FT‐ICR‐MS, (b) LC‐FT‐Orbitrap‐MS. DBE, double‐bond equivalent; NOSC, nominal oxidation state of carbon; KMD, Kendrick mass defect; NM, nominal mass.
Figure S3 Peak width normalised by maximal absolute intensity of the respective peak did not change the observed pattern in Figure 2.
Figure S4 (a,b) LC‐FT‐ICR‐MS, (c,d) LC‐FT‐Orbitrap‐MS peak width of shallow Southern Ocean (left column) and deep Southern Ocean (right column). The chromatographic peak width (1 min bins) of all molecular formulas of the Southern Ocean samples correlated with the number of heteroatoms and the signal intensity.
Figure S5 Coverage of PubChem stored isomer assemblages in the 50 most intense molecular formulas of DOM samples. Solid lines represent the density distribution of XlogP values, the dashed line represents the EIC of the DOM molecular formula (triangles depict the respective retention time bin of the LC‐FT‐Orbitrap‐MS).
Figure S6 Exemplary extracted ion chromatograms of the North Sea sample without smoothing shows broad elution of DOM molecular formulas. The mass trace was created with an accuracy of 0.9 ppm of the respective [M‐H]^−^ (C_17_H_22_O_8_ m/z 353.124224, C_18_H_22_O_8_ *m/z* 365.124072, C_19_H_22_O_10_ *m/z* 409.114105, C_22_H_28_O_12_ *m/z* 483.150750, C_23_H_30_O_11_ *m/z* 481.171471).
Figure S7 Full scan window cropped at *m/z* 850 at 5 min bin (top), 7 min bin (middle) and 9 min bin (bottom) of the North Sea sample, recorded with FT‐Orbitrap‐MS.
Figure S8 Selected scan windows between *m/z* 364.8 and *m/z* 365.3 at 5 min bin (top), 7 min bin (middle) and 9 min bin (bottom) of the North Sea sample, recorded with FT‐Orbitrap‐MS.
Table S1 Overview of all used standards. Substances were acquired from Merck.
Table S2 Spiked standard substances to the North Sea water sample. N. D., standard not detected.
Table S3 Statistics of weighted linear model (Figure 5). Left column for LC‐FT‐ICR‐MS, right column for LC‐FT‐Orbitrap‐MS. Slopes and intercepts are reported with confidence intervals.
Table S4 Molecular properties, intensities, retention time of 50 most intense molecular formulas detected by LC‐FT‐ICR‐MS.
Table S5 Molecular properties, intensities, retention time of 50 most intense molecular formulas detected by LC‐FT‐Orbitrap‐MS. The molecular formulas were consistent to the ones detected by LC‐FT‐ICR‐MS, Table S3.
Table S6 Number of structural motifs of the 50 most intense molecular formulas found in the respective structure data files.
Table S7: Statistics of the structural motifs of the 50 most intense molecular formulas corresponding to Figure 3. Distribution of the structural motifs are reported as median with 2.5% and 97.5% quantile.
Table S8 Retention time and XlogP values of pharmaceutical standards used for the linear regression.

**Data S1** Supporting information

**Data S2** Supporting information

## Data Availability

The binned peak list, and the annotated and filtered molecular formula list for both instruments have been submitted to the data publisher PANGAEA (https://pangaea.de/). As soon as curation and review are finished, the data will be made available.
