# Supplementary material for: From Retention Time to Functional Group Assignment: A Chemical Database‐Driven Approach for High‐Resolution Mass Data of Marine Dissolved Organic Matter
Source: Rapid Commun Mass Spectrom. 2025 Apr 14;39(13):e10043. doi: 10.1002/rcm.10043 (PMC11997241; doi:10.1002/rcm.10043)
Supplement: Supplementary file 1 — Figure S1 Screeplot of the principal component analysis, see Figure S2. Figure S2 Principal component analysis (PCA) of the weighted averages (wa) of the bulk parameters (weights: absolute intensity) for each sample and retention time bin. (a) LC‐FT‐ICR‐MS, (b) LC‐FT‐Orbitrap‐MS. DBE, double‐bond equivalent; NOSC, nominal oxidation state of carbon; KMD, Kendrick mass defect; NM, nominal mass. Figure S3 Peak width normalised by maximal absolute intensity of the respective peak did not change the observed pattern in Figure 2. Figure S4 (a,b) LC‐FT‐ICR‐MS, (c,d) LC‐FT‐Orbitrap‐MS peak width of shallow Southern Ocean (left column) and deep Southern Ocean (right column). The chromatographic peak width (1 min bins) of all molecular formulas of the Southern Ocean samples correlated with the number of heteroatoms and the signal intensity. Figure S5 Coverage of PubChem stored isomer assemblages in the 50 most intense molecular formulas of DOM samples. Solid lines represent the density distribution of XlogP values, the dashed line represents the EIC of the DOM molecular formula (triangles depict the respective retention time bin of the LC‐FT‐Orbitrap‐MS). Figure S6 Exemplary extracted ion chromatograms of the North Sea sample without smoothing shows broad elution of DOM molecular formulas. The mass trace was created with an accuracy of 0.9 ppm of the respective [M‐H]− (C17H22O8 m/z 353.124224, C18H22O8 m/z 365.124072, C19H22O10 m/z 409.114105, C22H28O12 m/z 483.150750, C23H30O11 m/z 481.171471). Figure S7 Full scan window cropped at m/z 850 at 5 min bin (top), 7 min bin (middle) and 9 min bin (bottom) of the North Sea sample, recorded with FT‐Orbitrap‐MS. Figure S8 Selected scan windows between m/z 364.8 and m/z 365.3 at 5 min bin (top), 7 min bin (middle) and 9 min bin (bottom) of the North Sea sample, recorded with FT‐Orbitrap‐MS. Table S1 Overview of all used standards. Substances were acquired from Merck. Table S2 Spiked standard substances to the North Sea water sample. [file RCM-39-e10043-s001.docx]

Supporting Information for

From retention time to functional group assignment: a chemical database-driven approach for high-resolution mass data of marine dissolved organic matter.

**Fabian Moye^1,2*^, Marlo Bareth^2,3^, Boris P. Koch^2,4^, Jan Tebben^2^, Tilmann Harder^1,2*^**

^1^Marine Chemistry, Faculty of Biology and Chemistry, University of Bremen, James Watt Str. 1, 28359 Bremen, Germany

^2^Department of Ecological Chemistry, Alfred-Wegener-Institut Helmholtz Zentrum für Polar- und Meeresforschung, Am Handelshafen 12, 27570 Bremerhaven, Germany

^3^Faculty of Mathematics and Computer Science, University of Bremen, Bibliothekstraße 5, 28359 Bremen, Germany

^4^University of Applied Sciences, An der Karlstadt 8, 27568 Bremerhaven, Germany

*Shared corresponding authors: Tilmann Harder (t.harder@uni-bremen.de), Fabian Moye (fmoye@uni-bremen.de)

## **Text S1.1.** Calculation of molecular formula parameter.

The nominal oxidation state of carbon (NOSC) was calculated according to LaRowe*, et al.* ^1^ with neutral charge and zero phosphorus:

$$NOSC=4-\frac{4C+H-3N-2O-2S}{C}$$

The aromaticity index was calculated according Koch*, et al.* ^2,^Koch*, et al.* ^3^:

$$AI=\frac{DBE_{AI}}{C_{AI}}=\frac{1+C-O-S-0.5(N+H)}{C-O-N-S}$$

The index of degradation (I_DEG_) was calculated according to Flerus*, et al.* ^7^ if all 10 molecular formulas were present: ($I_{abs}$: absolute intensity, NEG: with age negative correlating molecular formulas, POS: with age positive correlating molecular formulas):

$$I_{DEG}=\frac{\sum_{NEG} I_{abs}}{\sum_{NEG} I_{abs}+\sum_{POS} I_{abs}}$$

Similarly, the molecular formulas belonging to the terrestrial index (I*_terr_*, Medeiros*, et al.* ^8^) were identified and the index calculated if all 80 molecular formulas were present. A subset of reoccurring molecular formulas was used to create the I*_terr2_* (NEG: C_15_H_16_O_6_, C_17_H_16_O_7_, C_17_H_16_O_8_, C_17_H_18_O_7_, C_18_H_18_O_7_; POS: C_17_H_21_NO_8_, C_19_H_22_O_10_, C_20_H_24_O_10_, C_20_H_24_O_9_, C_20_H_26_O_9_). The index was only calculated for samples that contained all 10 molecular formulas:

$$I_{terr2}=\frac{\sum_{NEG} I_{abs}}{\sum_{NEG} I_{abs}+\sum_{POS} I_{abs}}$$

LC-FT-ICR-MS and LC-FT-Orbitrap-MS showed an overall overlap of 31.3 % annotated molecular formulas (2620). 4912 (58.7 %) formulas were uniquely detected by LC-FT-Orbitrap-MS while 937 (10.0 %) were unique to the LC-FT-ICR-MS dataset.

## **Text S1.2.** Multivariate Comparison

The Principal Component Analysis (PCA) of the intensity-weighted average bulk parameters showed for both mass spectrometers a separation of the retention time bins along the elution gradient in principal component PC1 (**Figure S2**). The LC-FT-ICR-MS data were also separated along PC2 by sample, whereas LC-FT-Orbitrap-MS data were not. The first two PCs explained most of the variance (LC-FT-ICR-MS: 89.13 %, LC-FT-Orbitrap-MS: 94.63 %, **Figure S2**). The molecular O/C ratio, the molecular N/C ratio, the DBE, the Kendrick mass defect (KMD), the NOSC, and the DBE/C ratio correlated positively with each other and decreased at later retention times. They correlated negatively with the molecular H/C ratio and the nominal mass (NM), which increased at later retention times (**Figure S2**).


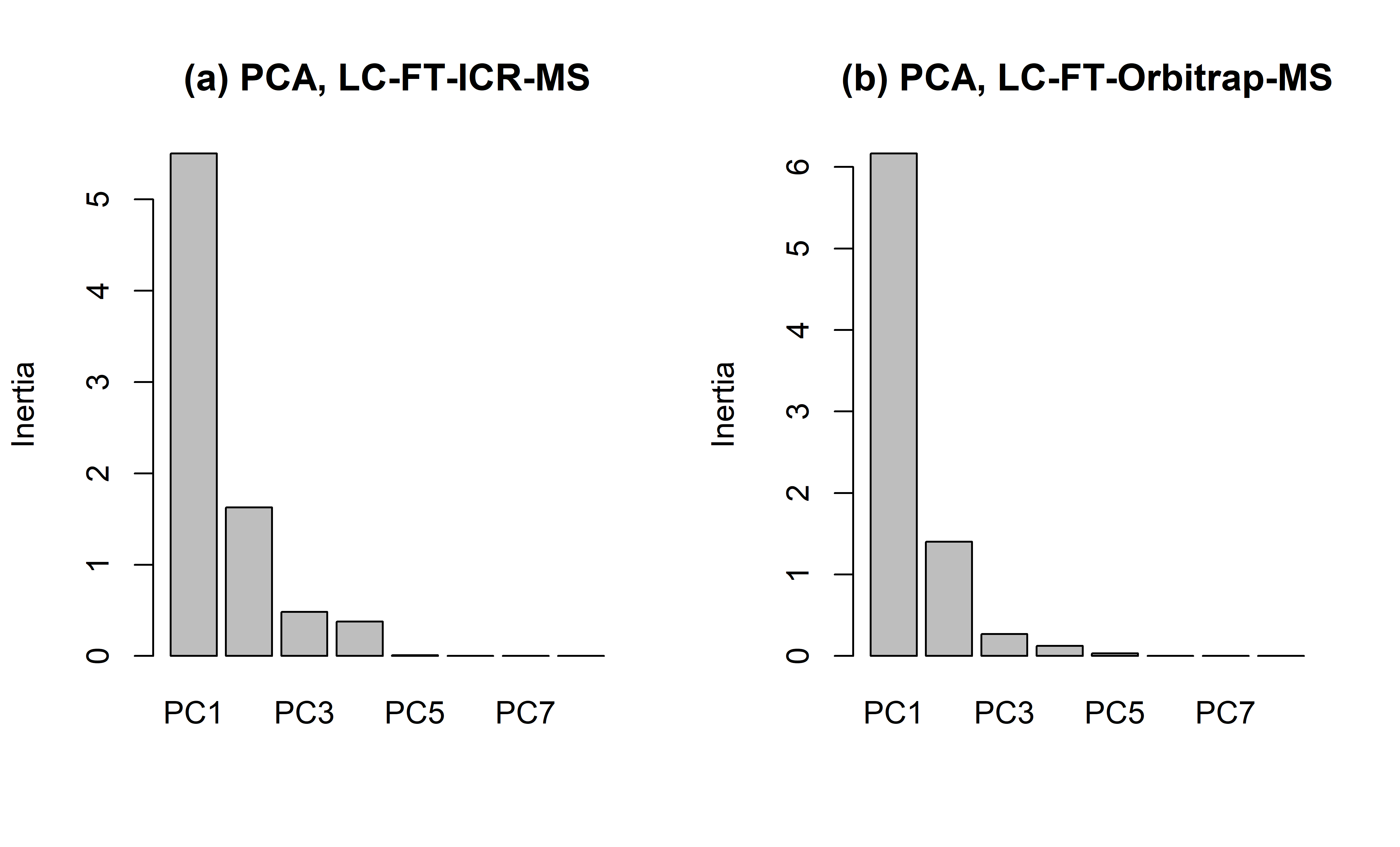


**Figure S1**: Screeplot of the principal component analysis, see **Figure S2**.


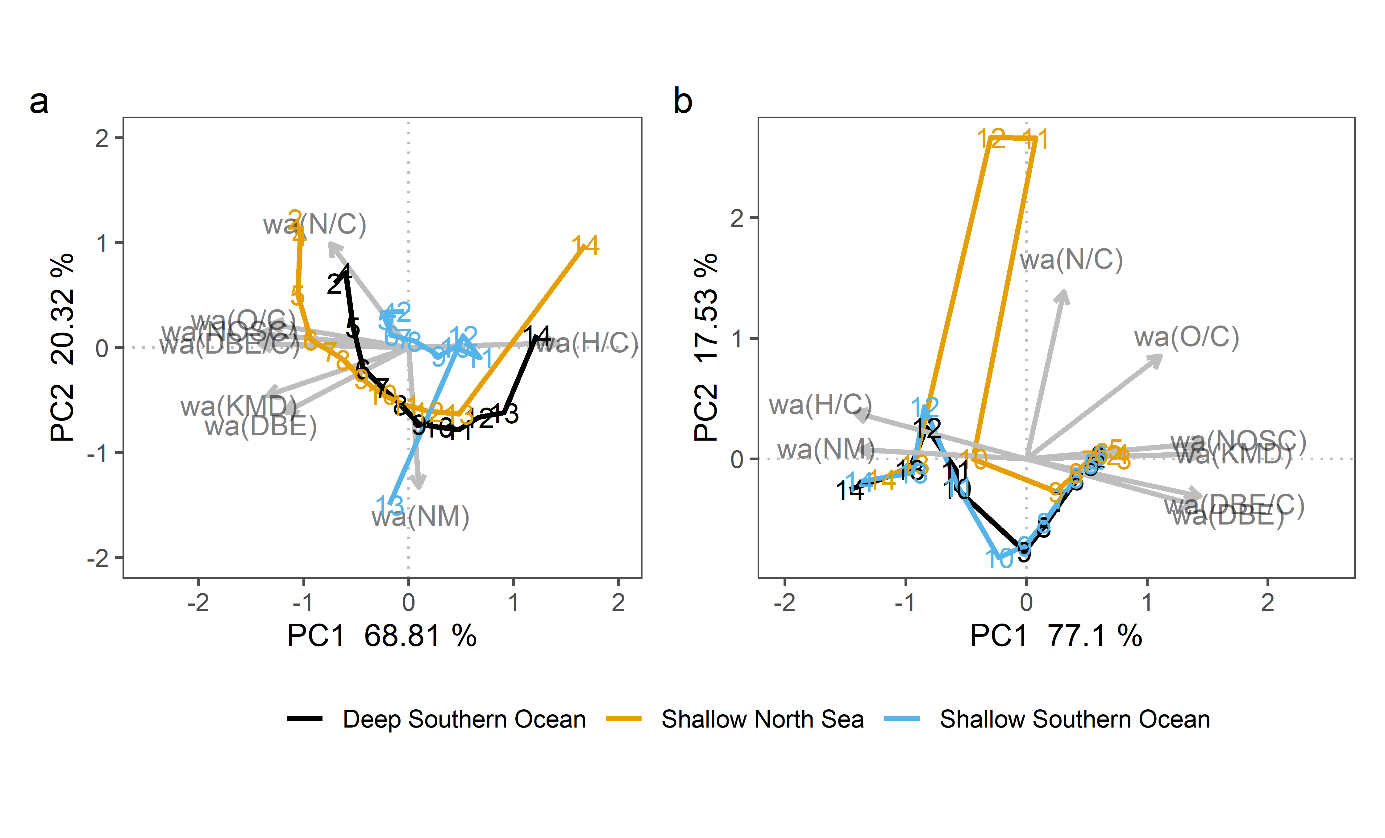


**Figure S2**: Principal component analysis (PCA) of the weighted averages (wa) of the bulk parameters (weights: absolute intensity) for each sample and retention time bin. (**a**): LC-FT-ICR-MS, (**b**): LC- FT-Orbitrap-MS. DBE: double bound equivalent, NOSC: nominal oxidation state of carbon, KMD: Kendrick mass defect, NM: nominal mass.


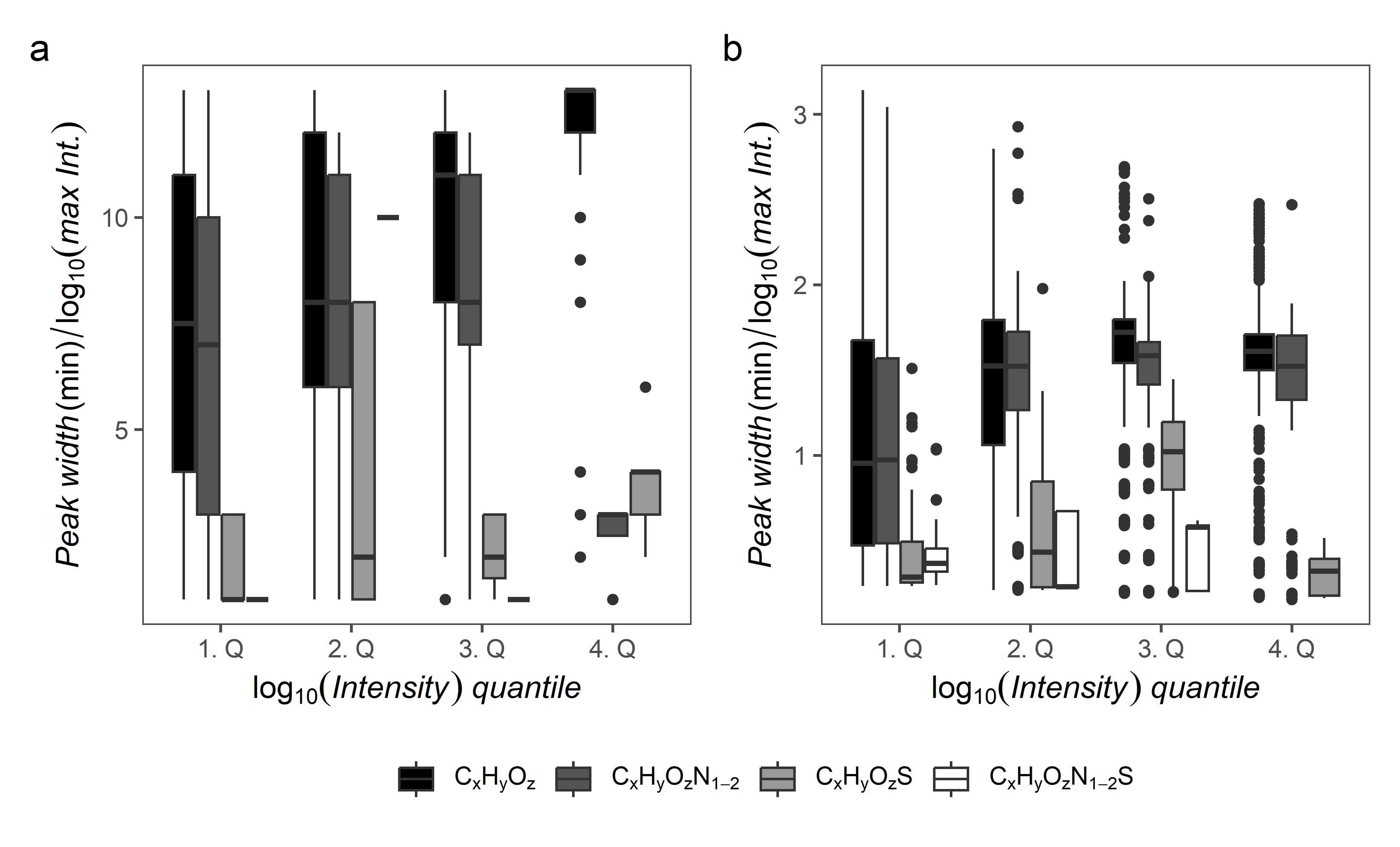


**Figure S3**: Peak width normalised by maximal absolute intensity of the respective peak did not change the observed pattern in **Figure 2**.


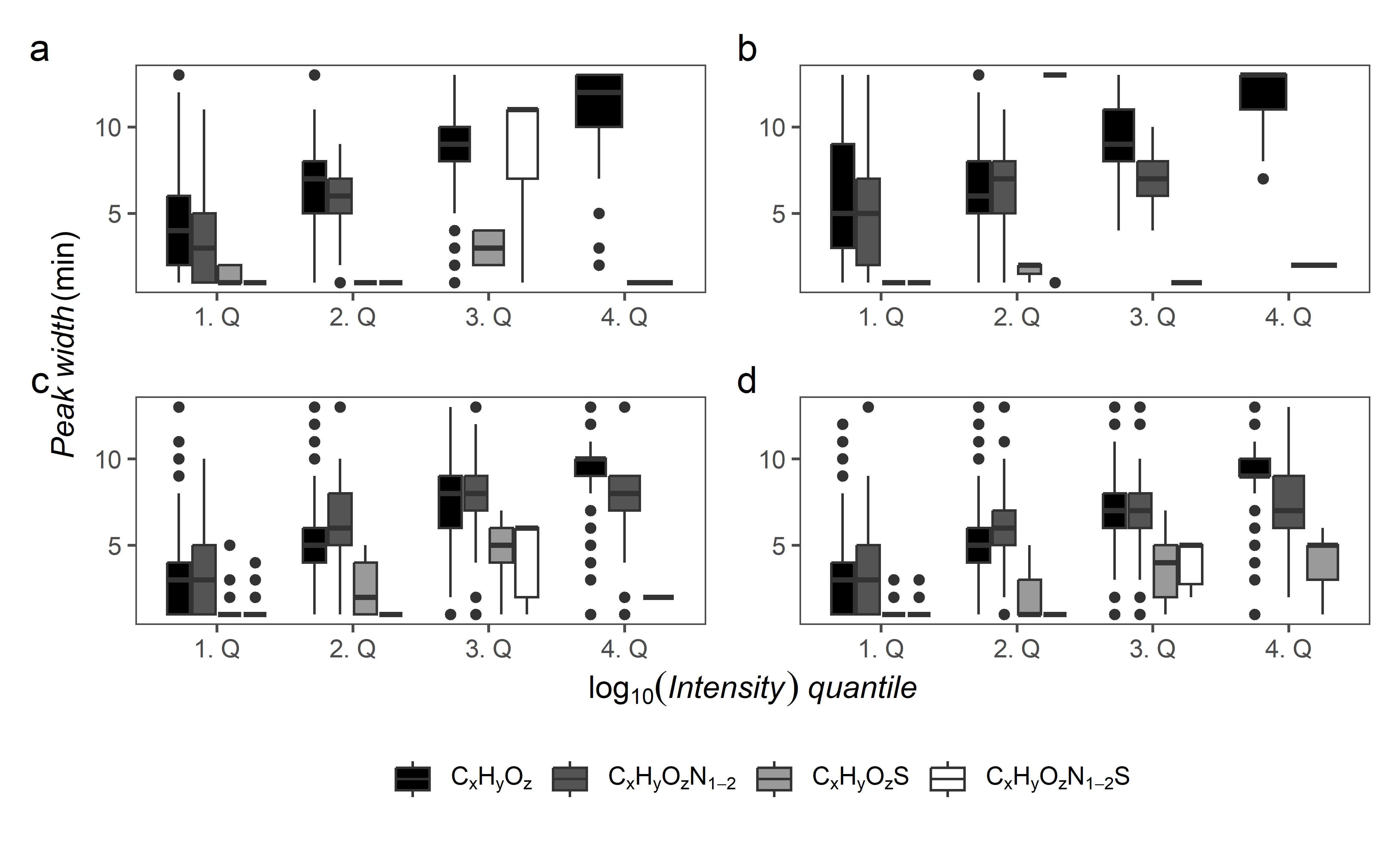


**Figure S4**: (**a, b**) LC-FT-ICR-MS, (**c, d**) LC-FT-Orbitrap-MS peak width of shallow Southern Ocean (left column) and deep Southern Ocean (right column). The chromatographic peak width (1 min bins) of all molecular formulas of the Southern Ocean samples correlated with the number of heteroatoms and the signal intensity.


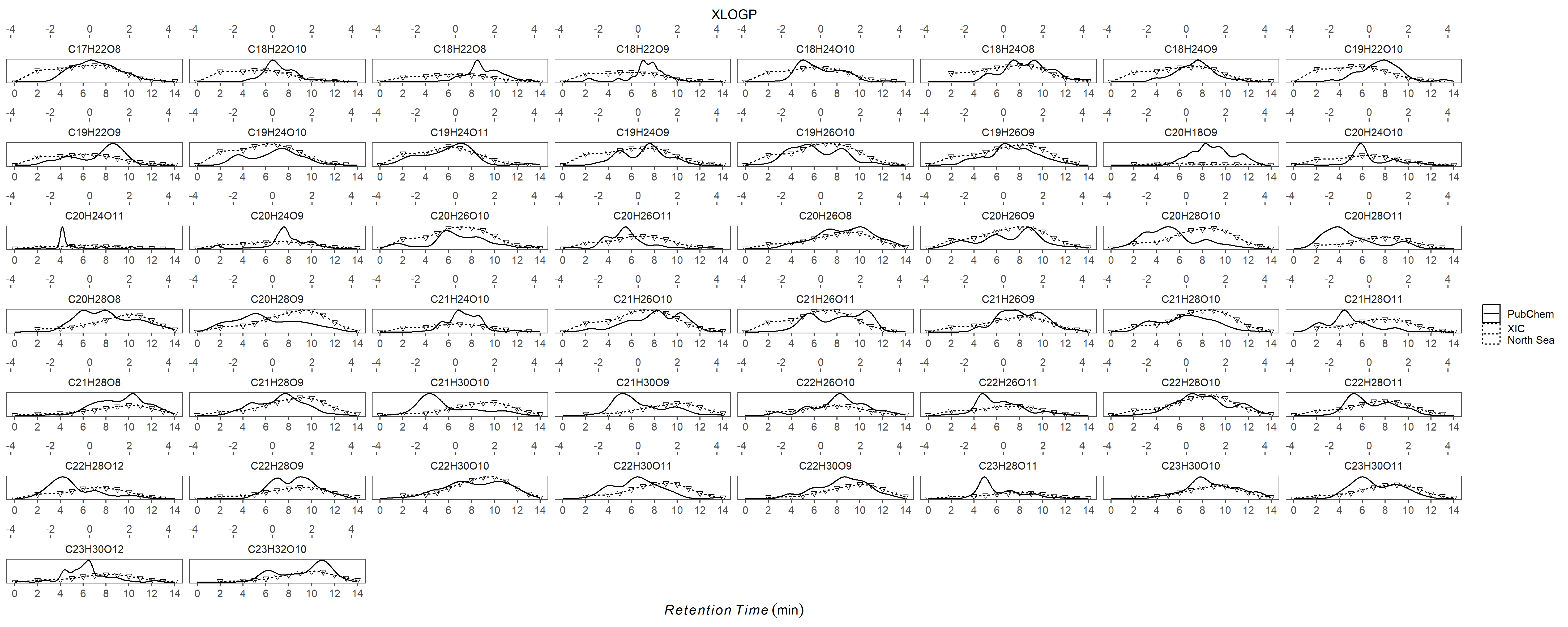


**Figure S5:** Coverage of PubChem stored isomer assemblages in the 50 most intense molecular formulas of DOM samples. Solid lines represent the density distribution of XLogP values, the dashed line represents the EIC of the DOM molecular formula (triangles depict the respective retention time bin of the LC-FT-Orbitrap-MS).

**
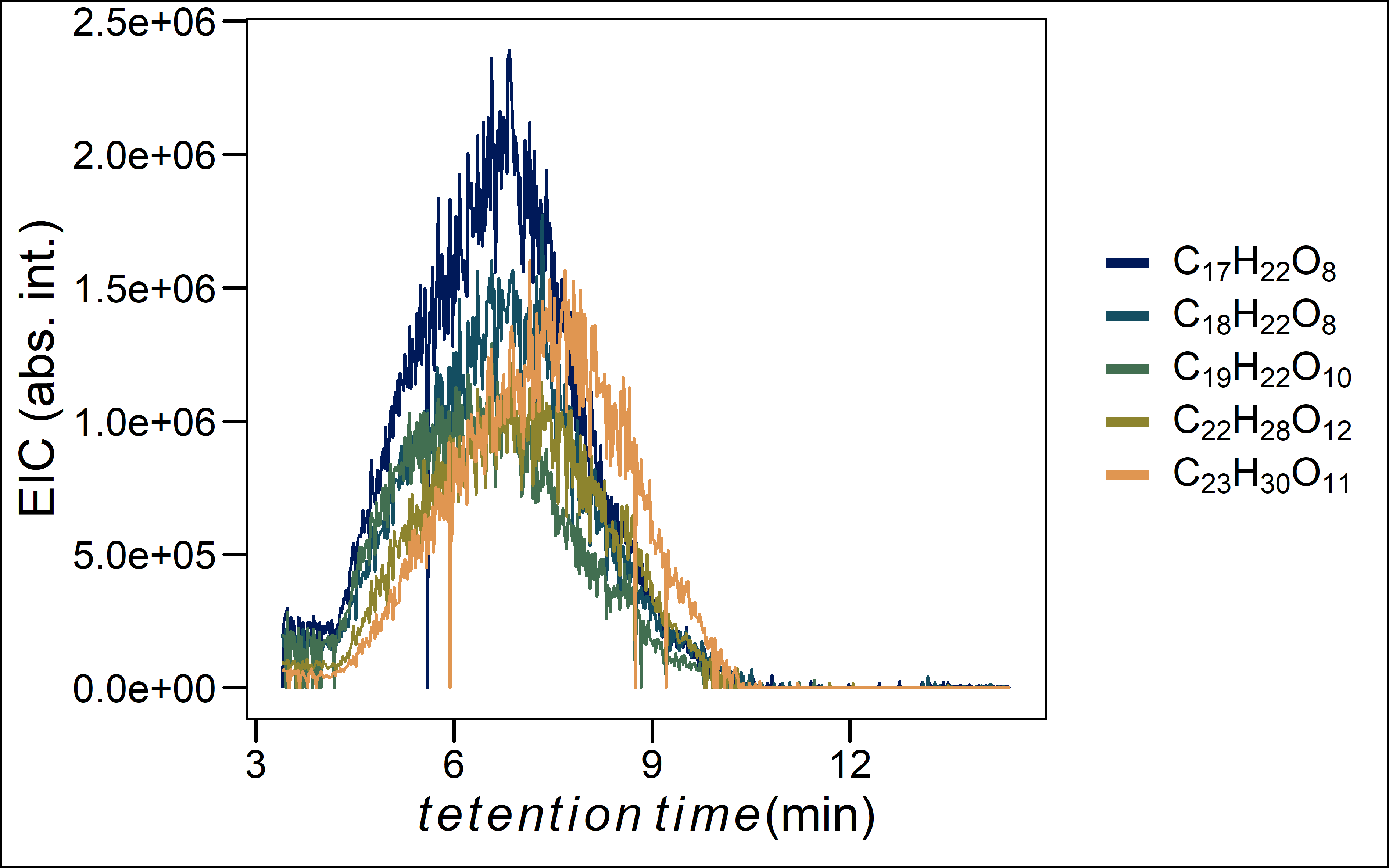
**

**Figure S6:** Exemplary extracted ion chromatograms of the North Sea sample without smoothing shows broad elution of DOM molecular formulas. The mass trace was created with an accuracy of 0.9 ppm of the respective [M-H]^-^ (C_17_H_22_O_8_ m/z 353.124224, C_18_H_22_O_8_ *m/z* 365.124072, C_19_H_22_O_10_ *m/z* 409.114105, C_22_H_28_O_12_ *m/z* 483.150750, C_23_H_30_O_11_ *m/z* 481.171471).

**Figure S7:** Full scan window cropped at *m/z* 850 at 5 min bin (top), 7 min bin (middle) and 9 min bin (bottom) of the North Sea sample, recorded with FT-Orbitrap-MS.

**Figure S8**: Selected scan windows between *m/z* 364.8 and *m/z* 365.3 at 5 min bin (top), 7 min bin (middle) and 9 min bin (bottom) of the North Sea sample, recorded with FT-Orbitrap-MS.

**Table S1**: Overview of all used standards. Substances were acquired from Merck.

| Standard | Product number | Molecular formula |
| --- | --- | --- |
| Aspirin | PHR1003 | C_9_H_8_O_4_ |
| Tylosin | BP349 | C_46_H_77_NO_17_ |
| Erythromycin | E5389 | C_37_H_67_NO_13_ |
| Tamoxiphen | 06734 | C_26_H_29_NO |
| Ranitidine | R0150000 | C_13_H_22_N_4_O_3_S |
| Tramadol | Y0000155 | C_16_H_25_NO_2_ |
| Paracetamol | P0300000 | C_8_H_9_NO_2_ |
| Salbutamol | S0100000 | C_13_H_21_NO_3_ |
| Phenoxymethyl penicilin | P1000000 | C_16_H_18_N_2_O_5_S |
| Valproat | S0930000 | C_8_H_15_NaO_2_ |
| Ibuprofen | PHR1004 | C_13_H_18_O_2_ |
| Triamterene | Y0000837 | C_12_H_11_N_7_ |
| Stiripentol | S6826 | C_14_H_18_O_3_ |
| Cimetidine | Y0001130 | C_10_H_16_N_6_S |
| Amitriptyline | Y000563 | C_20_H_23_N |
| Nicotine Ditatratdihydrat | Y0001425 | C_10_H_14_N_2_ |
| Clarithromycin | Y0000321 | C_38_H_69_NO_13_ |
| Noreistherone | N1200000 | C_20_H_26_O_2_ |
| Gabapentin Impurtiy D | Y0001349 | C_18_H_29_NO_3_ |
| Oseltamivir phosphate | SML1606 | C_16_H_28_N_2_O_4_ |
| Carbamazepine | 94496 | C_15_H_12_N_2_O |
| Lincomyacin hydrochlorid | 31727 | C_18_H_34_N_2_O_6_S |
| Lidocaine | BP727 | C_14_H_22_N_2_O |
| Cefuroxime sodium | C0695000 | C_16_H_15_N_4_NaO_8_S |
| Ketotifen | K2628-100mg | C_19_H_19_NOS |
| Caffeine | C051 | C_8_H_10_N_4_O_2_ |
| Gabapentine | G007 | C_9_H_17_NO_2_ |

**Table S2**: Spiked standard substances to the North Sea water sample. N. D.: Standard not detected.

| Mass (*m/z*) | Formula | Species | Name | Abs. Int. | RT (min) |
| --- | --- | --- | --- | --- | --- |
| 349.08637 | C16H18N2O5S | - H | Phenoxymethyl penicilin | 7.09E+06 | 7.95 |
| 395.09184 | C16H18N2O5S | + HCOO | Phenoxymethyl penicilin | 2.30E+04 | 7.97 |
| 306.20747 | C18H29NO3 | - H | Gabapentin Impurtiy D | 2.89E+03 | 8.25 |
| 352.21295 | C18H29NO3 | + HCOO | Gabapentin Impurtiy D | 1.88E+03 | 8.9 |
| 238.14487 | C13H21NO3 | - H | Salbutamol | 1.57E+07 | 6.92 |
| 284.15035 | C13H21NO3 | + HCOO | Salbutamol | 3.53E+04 | 6.92 |
| 251.10844 | C10H16N6S | - H | Cimetidine | 1.53E+07 | 7.72 |
| 297.11392 | C10H16N6S | + HCOO | Cimetidine | 1.41E+06 | 7.72 |
| 313.13398 | C13H22N4O3S | - H | Ranitidine | 2.10E+07 | 9.03 |
| 359.13946 | C13H22N4O3S | + HCOO | Ranitidine | 8.80E+05 | 9.02 |
| 311.19763 | C16H28N2O4 | - H | Oseltamivir phosphate | N.D. | N.D. |
| 357.20311 | C16H28N2O4 | + HCOO | Oseltamivir phosphate | N.D. | N.D. |
| 405.20648 | C18H34N2O6S | - H | Lincomyacin hydrochlorid | 4.69E+06 | 8.62 |
| 451.21196 | C18H34N2O6S | + HCOO | Lincomyacin hydrochlorid | 1.35E+07 | 8.62 |
| 205.1234 | C13H18O2 | - H | Ibuprofen | N.D. | N.D. |
| 251.12888 | C13H18O2 | + HCOO | Ibuprofen | N.D. | N.D. |
| 423.06161 | C16H16N4O8S | - H | Cefuroxime sodium | 2.26E+06 | 5.8 |
| 469.06709 | C16H16N4O8S | + HCOO | Cefuroxime sodium | N.D. | N.D. |
| 170.11865 | C9H17NO2 | - H | Gabapentine | N.D. | N.D. |
| 216.12413 | C9H17NO2 | + HCOO | Gabapentine | N.D. | N.D. |
| 732.45396 | C37H67NO13 | - H | Erythromycin | N.D. | N.D. |
| 778.45944 | C37H67NO13 | + HCOO | Erythromycin | 6.58E+05 | 11.35 |
| 193.0731 | C8H10N4O2 | - H | Caffeine | N.D. | N.D. |
| 239.07858 | C8H10N4O2 | + HCOO | Caffeine | N.D. | N.D. |

**Table S3**: Statistics of weighted linear model (Figure 5). Left column for LC-FT-ICR-MS, right column for LC-FT-Orbitrap-MS. Slopes and intercepts are reported with confidence intervals.

|  | LC-FT-ICR-MS | LC-FT-Orb.-MS |
| --- | --- | --- |
| (Intercept) | 0.48 *** | 0.61 *** |
|  | [0.46, 0.51] | [0.58, 0.63] |
| wa_xlogp | 0.29 *** | 0.48 *** |
|  | [0.27, 0.30] | [0.45, 0.51] |
| het[CHO]N | 0.42 *** | -0.03 |
|  | [0.37, 0.47] | [-0.07, 0.01] |
| het[CHO]S | 0.68 ** | 0.29 * |
|  | [0.26, 1.09] | [0.07, 0.51] |
| het[CHO]NS | 0.49 | 0.00 |
|  | [-0.47, 1.45] | [-0.41, 0.642] |
| sample_typeShallow SO | -0.19 *** | 0.34 *** |
|  | [-0.24, -0.14] | [0.30, 0.37] |
| sample_typeDeep SO | -0.06 ** | 0.35 *** |
|  | [-0.10, -0.02] | [0.32, 0.39] |
| wa_xlogp:het[CHO]N | -0.13 *** | -0.58 *** |
|  | [-0.17, -0.09] | [-0.63, -0.53] |
| wa_xlogp:het[CHO]S | -0.22 * | -0.30 *** |
|  | [-0.40, -0.04] | [-0.48, -0.13] |
| wa_xlogp:het[CHO]NS | -0.21 | -0.58 *** |
|  | [-0.66, 0.23] | [-0.82, -0.35] |
| wa_xlogp:sample_typeShallow SO | 0.50 *** | 0.39 *** |
|  | [0.48, 0.53] | [0.36, 0.43] |
| wa_xlogp:sample_typeDeep SO | 0.40 *** | 0.42 *** |
|  | [0.38, 0.43] | [0.38, 0.45] |
| het[CHO]N:sample_typeShallow SO | 0.46 *** | -0.01 |
|  | [0.36, 0.56] | [-0.05, 0.07] |

Cont. of Table S3.

|  | LC-FT-ICR-MS | LC-FT-Orb.-MS |
| --- | --- | --- |
| het[CHO]S:sample_typeShallow SO | -0.74 | 0.20 |
|  | [-2.06, 0.59] | [-0.12, 0.51] |
| het[CHO]NS:sample_typeShallow SO | -0.41 | -1.08 *** |
|  | [-1.80, 0.98] | [-1.64, -0.53] |
| het[CHO]N:sample_typeDeep SO | 0.33 *** | 0.01 |
|  | [0.25, 0.41] | [-0.05, 0.08] |
| het[CHO]S:sample_typeDeep SO | -0.60 | 0.25 |
|  | [-2.04, 0.84] | [-0.06, 0.57] |
| het[CHO]NS:sample_typeDeep SO | 1.23 | -1.05 *** |
|  | [-0.08, 2.53] | [-1.61, -0.48] |
| wa_xlogp:het[CHO]N:sample_typeShallow SO | -0.45 *** | 0.09 * |
|  | [-0.53, -0.37] | [0.02, 0.15] |
| wa_xlogp:het[CHO]S:sample_typeShallow SO | -0.51 * | 0.18 |
|  | [-0.96, -0.06] | [-0.06, 0.41] |
| wa_xlogp:het[CHO]NS:sample_typeShallow SO | -0.72 * | -0.08 |
|  | [-1.42, -0.03] | [-0.39, 0.22] |
| wa_xlogp:het[CHO]N:sample_typeDeep SO | -0.22 *** | 0.08 * |
|  | [-0.27, -0.16] | [0.01, 0.15] |
| wa_xlogp:het[CHO]S:sample_typeDeep SO | -0.34 | 0.08 |
|  | [-0.81, 0.13] | [-0.14, 0.31] |
| wa_xlogp:het[CHO]NS:sample_typeDeep SO | -0.97*** | -0.29 ** |
|  | [-1.50, -0.43 | [-0.60, -0.02] |
| N | 22278 | 46166 |
| R^2^ | 0.474 | 0.23 |
| *** p < 0.001; ** p < 0.01; * p < 0.05. | | |

Tables S4 to S8 are stored in the Excel sheet SI_tables.xlsx

**Table S4**: Molecular properties, intensities, retention time of 50 most intense molecular formulas detected by LC-FT-ICR-MS.

**Table S5**: Molecular properties, intensities, retention time of 50 most intense molecular formulas detected by LC-FT-Orbitrap-MS. The molecular formulas were consistent to the ones detected by LC-FT-ICR-MS, **Table S3**

**Table S6**: Number of structural motifs of the 50 most intense molecular formulas found in the respective structure data files.

**Table S7**: Statistics of the structural motifs of the 50 most intense molecular formulas corresponding to **Figure 3**. Distribution of the structural motifs are reported as median with 2.5 % and 97.5 % quantile.

**Table S8**: Retention time and XLogP values of pharmaceutical standards used for the linear regression.

References

1. LaRowe DE, Van Cappellen P. Degradation of natural organic matter: A thermodynamic analysis. *Geochimica et Cosmochimica Acta.* 2011;75(8):2030-2042. doi:10.1016/j.gca.2011.01.020.

2. Koch BP, Dittmar T. From mass to structure: an aromaticity index for high-resolution mass data of natural organic matter. *Rapid Communications in Mass Spectrometry.* 2006;20(5):926-932. doi:10.1002/rcm.2386.

3. Koch BP, Dittmar T. From mass to structure: an aromaticity index for high-resolution mass data of natural organic matter (vol 20, pg 926, 2006). *Rapid Communications in Mass Spectrometry.* 2016;30(1):250-250. doi:10.1002/rcm.7433.

4. Herzsprung P, Hertkorn N, von Tumpling W, Harir M, Friese K, Schmitt-Kopplin P. Understanding molecular formula assignment of Fourier transform ion cyclotron resonance mass spectrometry data of natural organic matter from a chemical point of view. *Anal Bioanal Chem.* 2014;406(30):7977-7987. doi:10.1007/s00216-014-8249-y.

5. Lechtenfeld OJ, Koch BP, Gasparovic B, Frka S, Witt M, Kattner G. The influence of salinity on the molecular and optical properties of surface microlayers in a karstic estuary. *Marine Chemistry.* 2013;150:25-38. doi:10.1016/j.marchem.2013.01.006.

6. Leefmann T, Frickenhaus S, Koch BP. UltraMassExplorer: a browser-based application for the evaluation of high-resolution mass spectrometric data. *Rapid Commun Mass Spectrom.* 2019;33(2):193–202. doi:10.1002/rcm.8315.

7. Flerus R, Lechtenfeld OJ, Koch BP, et al. A molecular perspective on the ageing of marine dissolved organic matter. *Biogeosciences.* 2012;9(6):1935-1955. doi:10.5194/bg-9-1935-2012.

8. Medeiros PM, Seidel M, Niggemann J, et al. A novel molecular approach for tracing terrigenous dissolved organic matter into the deep ocean. *Global Biogeochemical Cycles.* 2016;30(5):689-699. doi:10.1002/2015gb005320.
